# Progress in Orthopædic Surgery

**Published:** 1904-05-07

**Authors:** 


### Progress in Orthopædic Surgery.

(*Continued from Page* 85.)

Tubercular Disease of the Hip-joint.—A. H. Tubby[5] says that the most common deformity affecting the hip is tubercular coxitis. As a rule the disease appears before the ages of 15 or 18, and is rare after 30 or 40. Up to puberty it occurs with equal frequency in both sexes, but after that age it is more often seen in boys, girls leading a quieter life, and consequently being less exposed to injury. A history of traumatism is often given, but in all cases of the affection there is strong reason to suspect the existence of tubercular disease in some part of the body. In children in one-sixth to one-tenth of the cases the disease commences in the synovial membrane, and five-sixths or more in the bones, the most common site of onset being on either side of the epiphysial line of the neck of the femur. In other cases it commences on either side of the trochanteric epiphysial line, the course it takes depending on the point first affected and the treatment. When originating on the joint side of the epiphysis of the neck, affection of the joint almost invariably results, the head of the femur being sometimes cast off as a large sequestrum. If the disease progresses towards the anterior surface of the neck, it always spreads to the joint, but if it should make its way posteriorly, it may not enter the joint at all. Cases commencing in the trochanter are accompanied by symptoms resembling those of hip-joint disease, but in the absence of marked signs of this affection, the case is probably one of trochanteric epiphysitis. Infection of the joint and shaft of the femur may result, giving rise to tubercular diaphysitis. The disease may also originate in the acetabulum. With regard to the symptoms, constitutional signs precede local, the child getting out of health, becoming pale, losing appetite, and not sleeping well. There is a characteristic limp, more pronounced towards the end of the day, and pain, usually most marked near the hip-joint, and worse when the child moves about, may be present. When present in the knee this symptom often leads to mistakes in diagnosis. The deformities present in the early stage are abduction of the limb, rotation outwards, and apparent lengthening; in the second stage adduction, inversion, and apparent shortening. In some cases flexion and lordosis are marked, in others these are comparatively slight. Rigidity about the hip is the first local sign to appear, but there is sometimes great difficulty in eliciting it satisfactorily. Another sign, which Mr. Tubby considers reliable, is that if both limbs can be placed in the flexed and fully-abducted position hip-joint disease is not present. He believes that in one case in six to one in ten the disease is followed by abscess, which makes the prognosis much more unfavourable, abscess in this region being liable to become contaminated with septic material. The situation of the abscess depends on where the disease has broken down, and the way in which pus tracks through the muscles. In cases of pelvic abscess examination should be made per rectum. The resulting deformity may be either temporary or permanent. As a result of the inflammatory process all the ligaments become softened, and the writer has seen cases of sudden dislocation of the head of the femur occurring in connection with hip joint disease, followed by fibrous or bony ankylosis, causing permanent deformity of the joint. The affection must be distinguished from congenital dislocation of the hip, paralytic dislocation of the hip, coxa vara, sacro-iliac disease in the early stage, and osteitis in the great trochanter. In children the disease has sometimes been mistaken for appendicitis on the right side, and especially so in cases where a pelvic abscess has been present. The diagnosis is often difficult, especially in reference to coxa vara. Uncomplicated cases require three years of treatment, and possibly longer, there being a great tendency for these cases to relapse. The mode of treatment recommended is by rest and weight-extension, the weight applied being two and a half pounds for a child of three years of age, with an increase of half a pound for every year beyond this up to five or six pounds. If an abscess be present, it should be opened, the cavity thoroughly cleansed, and sewn up immediately, there being great risk of streptococcic and staphylococcic infection following. Mr. Tubby prefers Adams's operation to Gant's infra-trochanteric osteotomy for the treatment of severe deformity of the hip joint. He is opposed to excision of the hip as a formal operation, and says that the cases in which amputation is indicated have greatly diminished, owing to improved methods of treatment. Menciere[6] advocates a more direct attack on the tuberculous lesions in the early stage, and anticipates good results from an extension of the method recommended by Watson Cheyne and Phelps, consisting in the use of pure carbolic acid in the treatment of old tuberculous sinuses and advanced joint disease. In the early stage he advises rest and frequent injections of iodoform (in ether) into the joint and peri-articular tissues, with the injection of pure carbolic into the bones. A puncture is made in the tissues, extending down to the bone, a small drill introduced into the epiphysis, a metal pipette or tube inserted into this opening, and pure carbolic introduced into the bone without coming in contact with the other tissues. The carbolic is allowed to remain in for one minute, then mopped up by inserting pieces of cotton-wool into the tube, after which the part is washed out with absolute alcohol. In the more advanced cases an open incision is made down to the bone, and a larger hole drilled in the epiphysis, which is treated as above. In further advanced cases he performs complete excision, washing out the wound with carbolic acid, followed by alcohol, according to the method practised by Phelps.

Treatment of Advanced Tuberculous Disease of the Knee-joint.—In reply to a set of inquiries on this subject, sent by Professor Wright and Mr. W. F. Haslam [7] to hospital surgeons, attached to various medical schools in the United Kingdom, the opinions expressed were as follows :—As regards the indications for operation, the general view appears to be that progressive disease, in spite of adequate treatment, especially when associated with suppuration or commencing dislocation, calls for this method of treatment. The majority prefer erasion to excision in young patients with early disease, but a like majority would excise in adults, with advanced disease. In erasion, the horseshoe incision, with division of the ligament or of the patella, seems to be the generally approved method, and opinion is strongly in favour of preserving the patella in this operation. A large majority also preserve the crucial ligaments if possible. There is no great difference of opinion as to the treatment of bone lesions, nearly all scooping, gouging, or scraping out local foci. A considerable number apply some antiseptic—iodoform, carbolic acid, or zinc chloride. Opinion is rather in favour of the use of the tourniquet, removing it before applying the dressings. Drainage is dispensed with in most cases by the third day, opinion being fairly evenly divided as to its advantages and disadvantages. Twenty-six out of 65 surgeons use aseptic dressings, the rest use some form of antiseptic. There is considerable difference of opinion with regard to the best form of splint, but a back splint in some form is used by most surgeons, the time it is kept on varying from 10 days to five years. The belief that flexion occurs after operation is almost unanimous, and prolonged fixation by leather, poroplastic, or Thomas's splint is the generally accepted means of preventing it. When the union is fibrous, various methods of dealing with flexion are recommended ; in osseous union there is little difference of opinion. The great majority of those practising erasion make no attempt to obtain a mobile joint afterwards, and the most opposite beliefs are expressed in regard to comparative shortening in erasion and excision. Twenty-five surgeons are of opinion that the primary seat of the disease is usually synovial, five that it is only synovial, 15 that it begins most commonly in bone, one always in bone, 18 think it may start in either tissue. Mention is also made of disease beginning in the perisynovial cellular tissue, and the occurrence of latent bone disease is noticed. Professor Wright considers that, after three months' treatment of tuberculous knee, with no resulting improvement, or with evidence of caseation, operation should be undertaken, but that the great majority of cases can be cured without operation, if the patient is seen at an early stage of the disease. He does not perform excision if erasion is possible, and does not perform erasion if the local disease is so far advanced that it is unremovable, or the patient's health is broken down, so as to preclude a long operation. In erasion he employs the transverse transpatellar incision, with a vertical upward incision occasionally added. He uses an elastic tourniquet, sometimes removed before dressing, sometimes not, and drains the wound only if it is known to be septic. The dressings employed are sublimate or biniodide gauze and wood-wool wadding. The limb is put up in a wooden back splint, with two side splints, for about three weeks, then in a Thomas's knee-splint, or a Paul's splint with plaster of Paris, preferably Paul's splint, and the use of a Thomas's knee-splint is recommended for at least two years. If flexion occurs, gradual extension or elastic pressure is employed : if this fails, forcible straightening under chloroform ; if there is backward displacement, the joint is opened up, excising if necessary. When there is osseous union, osteotomy is performed, with or without the removal of a wedge from the femur above the joint. After erasion shortening of the limb does not occur in the majority of cases and when it does is probably due to premature synostosis of the epiphysial line or disuse of a flexed limb. Professor Wright performs excision in a child only when compelled to do so, and prefers erasion in adults if possible. He uses no direct fixation after excision, and leaves the patella unless it is diseased. He expresses the opinion that the great majority of cases of tuberculosis of the knee are probably synovial in origin and that the presence of latent foci in the bone is exceptional. He thinks that erasion would be more in repute if it were always properly performed, failure being frequently attributable to the fact that the disease is not entirely removed.

[5] Clin. Jour., June 10, 1903, p. 113. [6] Arch. Prov. de Chirurgie, Oct. 1, 1902. [7] Brit. Med. Jour., Oct. 10, 1903, p. 888.

*(To be continued.)*

